# 

**Published:** 1842-07

**Authors:** 


### WESTERN JOURNAL

OF

### MEDICINE AND SURGERY.

### EDITORS.

---

DANIEL DRAKE, M. D.,

PROFESSOR OF PATHOLOGICAL ANATOMY AND CLINICAL MEDICINE
IN THE MEDICAL INSTITUTE OF LOUISVILLE:

LUNSFORD P. YANDELL, M. D.,

PROFESSOR OF CHEMISTRY AND PHARMACY IN THE SAME:

THOMAS W. COLESCOTT, M. D.

### RECEIPTS FOR THE MEDICAL JOURNAL,

FOR JUNE, 1842.

| | |
|---|---|
| Dr. J. Wilcox, Williamsburg, O., - - - | $5 00 |
| W. R. Snelson, Fayette, Mo., - - - | 5 00 |
| W. J. S. Cornett, Versailles, Ia., - - | 10 00 |
| Bennett, Withamsville, O., - - - | 5 00 |
| M. D. Means, Preston, Miss., - - - | 4 00 |
| W. G. Montgomery, Independence, Ia., - - | 5 00 |
| Sale, Lebanon, Tenn., - - - - | 5 00 |
| Norman, Cedar Grove, Tenn., - - - | 5 00 |
| J. S. Helms, Tuscumbia, Ala., - - - | 5 00 |
| J. A. Green, Covington, Tenn., - - - | 5 00 |
| J. Travis, Martin's Creek, Tenn., - - | 5 00 |
| J. Scales, Wesley, Tenn., - - - | 5 00 |
| A. M. Clemens, Huntsville, Ala., (postage 38), - | 10 00 |
| W. P. Golliday, Farmington, Ill., - - | 2 00 |
| R. C. Price, Murfreesboro', Tenn., - - | 5 00 |
| N. F. Scales, Lewisburgh, Tenn., - - | 5 00 |
| A. D. Cage, Mt. Henry, Tenn., - - - | 5 00 |
| W. D. Dorris, Nashville, do (postage 38 cents), | 10 00 |
| J. T. Underwood, New Castle, Ky., - - | 7 30 |
| J. M. Adams, Somerville, O., - - - | 10 25 |
| J. D. Sale, Moscow, Tenn., - - - | 5 00 |
| F. Taylor, Westport, Ky., - - - | 5 00 |
| A. W. Scales, Harrodsburgh, Ky., - - | 5 00 |
| W. B. Townsend, Pleasant Hill, Ala., (dis. 60 cents), | 3 00 |
| C. Hatch, Benton, Miss., - - - - | 5 00 |
| E. Clifford, Grove's P. O., Ia., - - - | 5 00 |

### DELINQUENT LIST.

Below we commence a list of those subscribers, who having taken this Journal from its commencement, have not yet made a single payment. The main design of publishing this list is, to bring it forcibly to the minds of subscribers that they have not paid. Upon this hint, of course every one of them that has the least regard for his honor, will remit the amount he owes, under the post-office frank. In our next, we shall complete the list of this class of delinquents. In the succeeding number, we shall publish the whole of such as do not speak on this hint; and shall keep the list standing, striking off from time to time such as pay up. In two or three months the honest will be pretty effectually winnowed from the dishonest, and those who remain on the list will have a diploma which will distinguish them far beyond any they may have received from learned institutions.

In two or three months we shall commence a list of all those who may fail to remit after their accounts have been presented through the post office. In due time the public will be furnished with a very ready means of ascertaining the character of western physicians.

TENNESSEE.

J. Drake, South Carroll.
J. J. Gibson, Hampshire.
Wilson & Bearden, Petersburgh.
L. M. N. Cook, Silver Spring.
T. J. Walton, Mulloy's.
S. B. Robinson, Versailles.
J. M. Dean, Lynchburgh.
A. F. Clement, Lexington.
W. H. Warren, do
W. T. Eatherty, Rural Hill.
J. A. Lecky, Ripley.
S. Richardson, Rutherford.
W. D. Lee, do.
W. J. J. Morrow, Chattawogga.
J. W. Koen, Colliersville.
J. M. M. Cornelius, Germantown.
Robinson & Hagan, Carthage.
J. B. Copeland, Nolandsville.
R. Forbis, Elkton.
M. W. O'Brien, Springfield.
W. J. C. Rogers, Murfreesboro'.

NEW YORK.

Dr. Wm. Welch, Penfield.
Dr. G. Standing, Utica.

ARKANSAS.

W. A. Threlkeld, Helena.
J. H. McCarty, Ozark.
Dr. Beach, Lawrenceville.
J. Eaton, Pine Bluff.

LOUISIANA.

B. B. Smith, Shreveport.
J. B. Henderson, New Orleans.
J. Davis, Millckin's Bend.
T. Ingersoll, Cherryville.
E. Ragan, Columbia.

ALABAMA.

W. E. Murphy, Somerville.
P. L. Gilbert, Isny, Washington Co.
R. L. Graves, Leesville.
D. W. Ford, Meridianville.
E. M. Hussey, Waterloo.
W. R. Davie, do
W. F. Johnson, Benton.
J. Durno, Mobile, or Alton, Ill.
J. C. Harris, Cedar Bluff.
J. T. Reese, Syllacogga.

MISSISSIPPI.

J. B. Scott, Oakland.
Harris & McLaen, Wahallock.
S. Cook, Hernando.
E. F. Bouchelle, Columbus.
S. D. Kennedy, Holmesville.
D. B. Nailer, Vicksburgh.
—— Hogan, Panola.
A. P. Reed, Fort Adams.
J. A. Hodges, Garlandsville.
O. S. Echols, Hamilton.
L. Shelby, Warrenton.

ILLINOIS.

O. Abbey, Quincy.

W. H. Efner, Lacon.

J. W. Chenoweth, Auburn.

C. L. Duncan, Marshall.

J. R. Colwell, Farmington.

J. Kirkpatrick, Fox P. O.

W. Allison, Paradise.

W. Patton, Oakland.

—— Adams, Prarieton.

T. J. Beckwell, Rushville.

D. S. Smith, Juliet.

J. Jayne, Carlinsville.

B. F. Edwards, Alton.

C. F. Falley, York.

H. Symmes, Equality.

W. W. Higgins, Jones' P. O.

W. O. Chamberlain, Princeton.

Wood & Goodwin, Holland's Grove.

F. Canterbury, Cole's C. H.

H. P. Griswold, Plymouth.

J. M. Metcalf, Winchester.

W. Kellar, Decatur.

